# Variations in the Branching Pattern of the Aortic Arch Among Adults and Its Clinical Correlations: A Computed Tomographic Angiographic Study

**DOI:** 10.7759/cureus.88120

**Published:** 2025-07-16

**Authors:** Yashu Bhardwaj, Brijendra Singh, Rashmi Malhotra, Rahul Sharma, Pankaj Sharma

**Affiliations:** 1 Anatomy, All India Institute of Medical Sciences, Rishikesh, IND; 2 Radiology, All India Institute of Medical Sciences, Rishikesh, IND

**Keywords:** aortic arch, brachiocephalic trunk, common carotid artery, computed tomography, variations

## Abstract

Background: Variations can occur in the branching pattern of the aortic arch (AA) because of its complex embryological development. These variations are quite common and typically asymptomatic but can sometimes cause dyspnea, dysphagia, and intermittent claudication, as well as confusion in the interpretation of radiological scans.

Methods: In the present study, we analyzed the types and incidence of AA variations in 105 patients after careful computed tomographic angiographic (CTA) imaging.

Results: In the present study, we found that out of the 20 patients, three with variations in the branching pattern of the AA had experienced dyspnea (15%). The left common carotid artery (CCA) originated from the brachiocephalic trunk (BCT) in eight out of 20 patients (40%). The left vertebral artery (VA) originated from the AA in five (25%) patients. The thyroid ima artery (TIA) originated from the BCT in two participants (10%). The left CCA and BCT originated from the AA as a single trunk in two patients (10%). The left VA originated from the AA and the right VA from the BCT in one patient (5%). The BCT originated as the left branch from the AA in one patient (5%). The right and left CCA originated as a single trunk from the AA in one patient (5%).

Conclusion: The present prospective study is the first conducted in the Himalayan belt of North India. The findings highlight the importance of spreading awareness about variations in the branching pattern of the AA. This awareness is useful when executing endovascular procedures and planning cardiothoracic surgeries.

## Introduction

The aortic arch (AA) has three main branches. They are, from left to right, the left subclavian artery (LSA), the left common carotid artery (LCCA), and the brachiocephalic trunk (BCT). The BCT gives rise to the right subclavian artery (RSA) and the right common carotid artery (RCCA), and the vertebral arteries (VAs) originate from each subclavian artery. Because the formation of the AA and its branches during embryogenesis is complicated, deviations from the normal branching patterns are quite common and, typically, asymptomatic, becoming apparent only when a patient undergoes imaging, surgery, or autopsy [1].

Because of aberrant fetal AA involutions, the anatomical abnormalities of the AA typically manifest in the first and second trimesters. Six pairs of AAs arise from the aortic sac, which connects the ventral and dorsal aorta, and a fifth pair vanishes during fetal development. However, in some cases, the fifth arch persists and is known as the persistent fifth aortic arch (PFAA). The remaining AAs divide into distinct arteries that supply the lungs, upper limbs, head, and neck region. The adult AA is typically formed from the remaining left fourth AA with its usual branches [2].

Numerous chromosomal anomalies have been associated with anatomical variation in the AA branches. At least one congenital heart abnormality is present in up to 98.4% of pediatric patients with bovine AA. [1] The reported incidence of up to 35% among those with Down syndrome indicates that an anomalous RSA is a biomarker for the syndrome and its associated cardiac problems [1], though the RSA has not been confirmed as a biomarker for trisomies in large cohort studies [1].

More population-based morphometric investigations are necessary, even though anatomical variations are only found in a small percentage of cases, because of the significant positive impact in these cases and the potential for regional and ethnic variations [2]. The previous literature provides no data about AA branching patterns in the Himalayan belt of North India. Accordingly, the purpose of this study is to characterize the variations in these patterns to provide useful information for anatomists, radiologists, vascular surgeons, and neck and thoracic surgeons in North India. Though most of these anatomical variants are asymptomatic, a few can result in dyspnea, dysphagia, or intermittent claudication or complicate the interpretation of radiological scans or neck and thoracic surgery. Thus, insights into these variants can be helpful when surgeons breach the AA and its branches.

## Materials and methods

The present study was conducted prospectively in the Department of Anatomy, in collaboration with the Department of Radiodiagnosis, at the All India Institute of Medical Sciences, Rishikesh, Uttarakhand, India, over an 18-month period, from June 15, 2021, to December 15, 2022. The study included 105 participants, of whom 56 were male and 49 were female; all were in the age group 18 to 85 years and had undergone computed tomographic angiography (CTA). Out of the 105 participants, 20 had variations in the branching pattern of the arch of the aorta.

The inclusion criteria required data from adult patients who had undergone CTA from a 64-slice and 128-slice CT scanner, and high-quality CTA scans with sufficient resolution to assess the aortic arch and its branches accurately. Patients who had undergone previous CCA occlusion or had known vascular abnormalities (i.e., carotid hypoplasia, plaques in carotid bifurcation, or aneurysms in the neck or thoracic vessels) were excluded. The obtained images were evaluated and analyzed for variations in the AA. The statistical analysis was done using the chi-square test and Fisher’s exact test.

The study received approval from the Institutional Ethics Committee (IEC) of the All India Institute of Medical Sciences, Rishikesh (approval no. AIIMS/IEC/21/323). The CTA data was collected from a 64-slice and 128-slice CT scanner in the Department of Radiodiagnosis. The radiological data were collected consistently with the prefixed inclusion and exclusion criteria. We reconstructed the three-dimensional view of the CT images using a RadiAnt Dicom Viewer (Medixant, Poznan, POL) with software installed on an MSI laptop (Micro-Star International, New Taipei City, TWN). The multiplanar reconstruction (MPR) function was used to reconstruct the images in the orthogonal planes (i.e., coronal, sagittal, axial, or oblique, depending on the base image plane). This procedure enabled us to develop a new understanding of the anatomy that would not have been possible with the basic 2D photographs. A coronal series was then produced from more than 2,000 axial CT slices in about 3 seconds using the rapid reconstruction procedure (with a modern Intel Core i7 system). With the help of the 3D volume-rendering (VR) tool, contemporary CT scanners produce an enormous amount of data in three dimensions. The various components of the dataset were interactively explored in the 3D VR window.

The undesirable portions of the volume were removed using the scalpel tool in the software, thereby changing the volume, altering the zoom level and position, adjusting the color and opacity, and measuring the length to reveal the hidden structures. This freely downloadable software, installed on the MSI laptop, features the user-friendly measurement tools of multiplanar reconstruction and 3D VR. After the observation and evaluation, the images were obtained using these features to identify variations in the branching pattern of AA.

## Results

**Figure 1:**
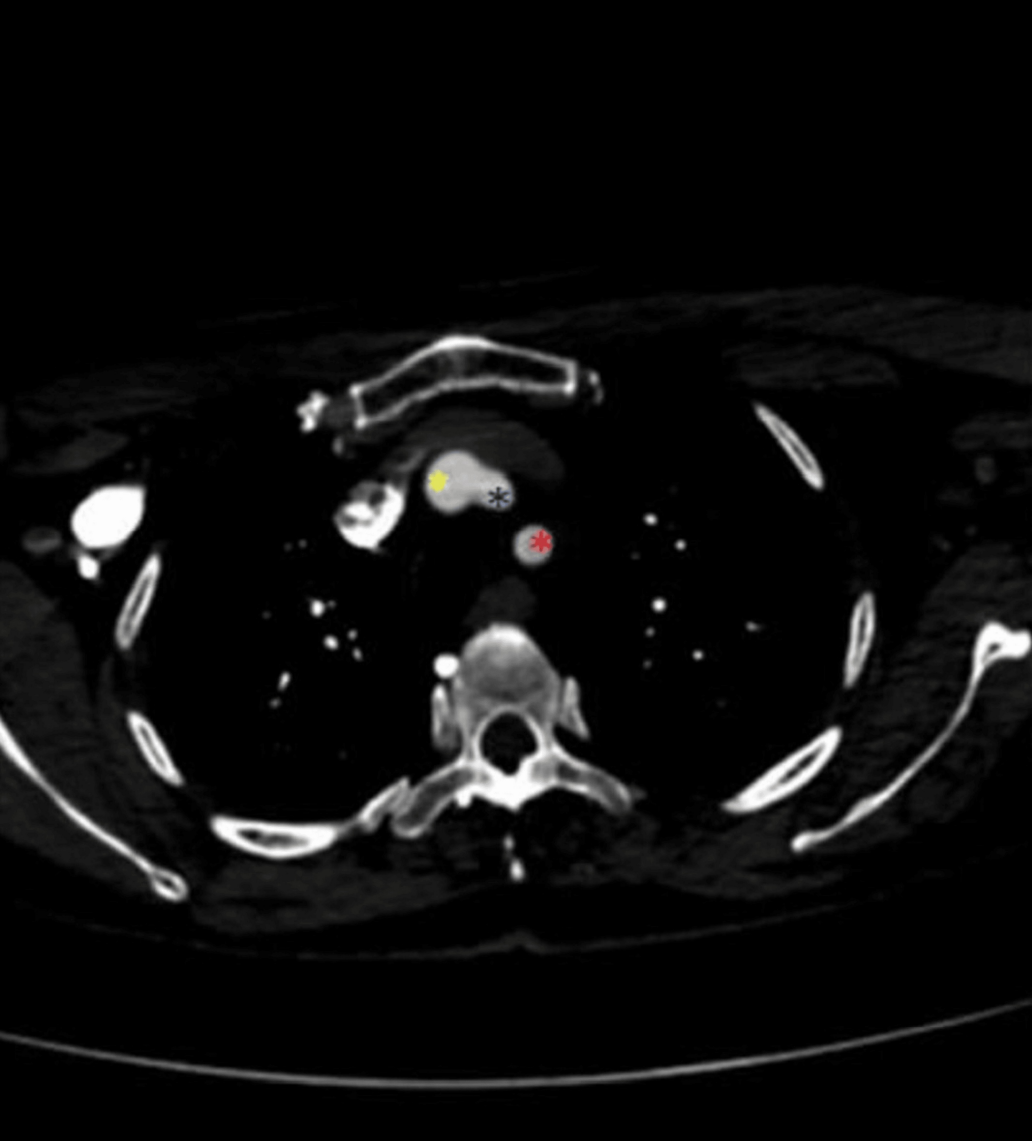
The LCCA originates from the BCT (axial view) The yellow asterisk marks the BCT, the dark blue asterisk marks the LCCA, and the red asterisk marks the LSA. LCCA: Left common carotid artery, BCT: Brachiocephalic trunk, LSA: Left subclavian artery

**Figure 2:**
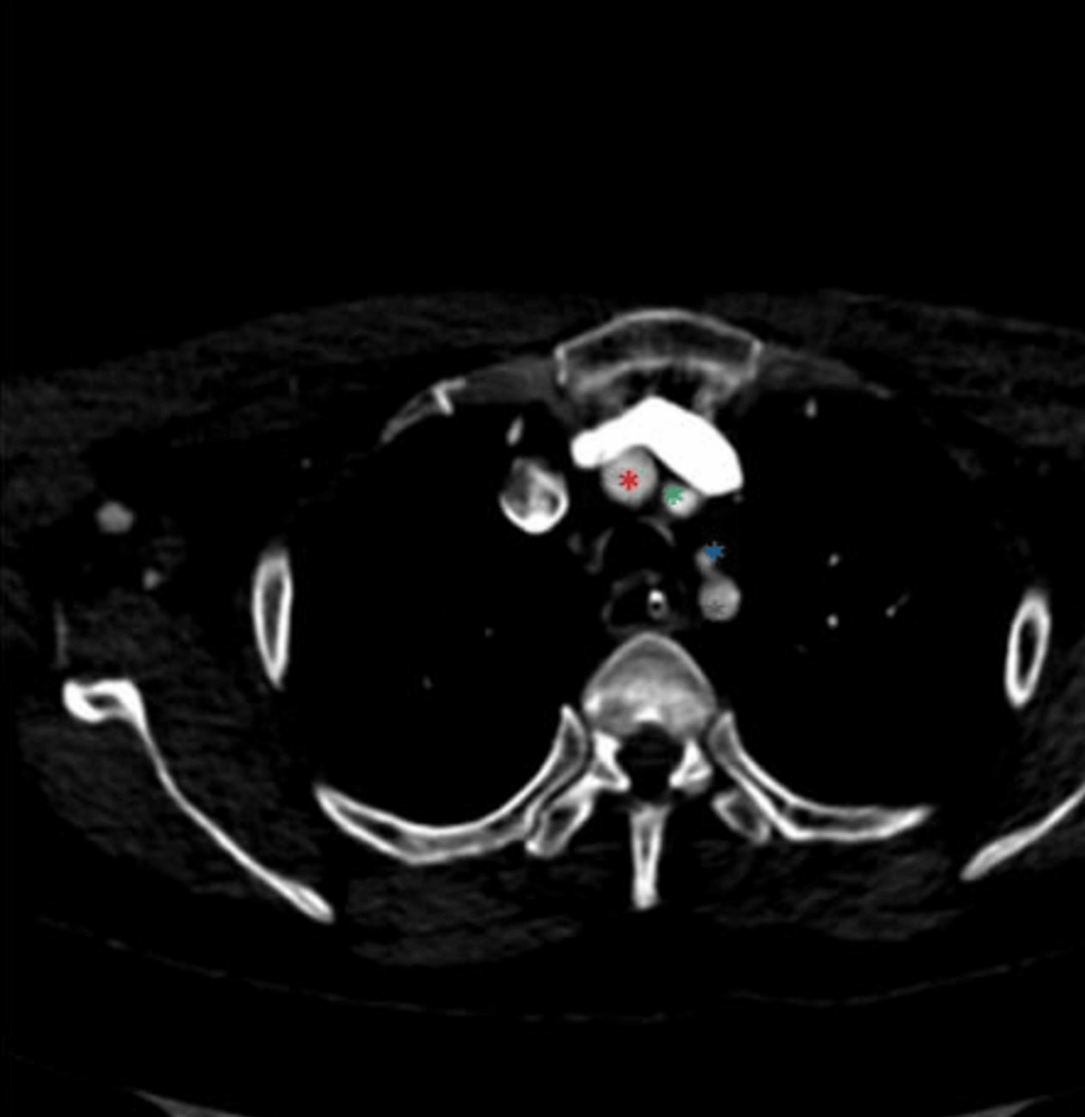
The LVA originates from the AA (axial view) The red asterisk marks the BCT, the green asterisk marks the LCCA, the blue asterisk marks the LVA, and the grey asterisk marks the LSA. LVA: Left vertebral artery, AA: Aortic arch, BCT: Brachiocephalic trunk, LCCA: Left common carotid artery, LSA: Left subclavian artery

**Figure 3:**
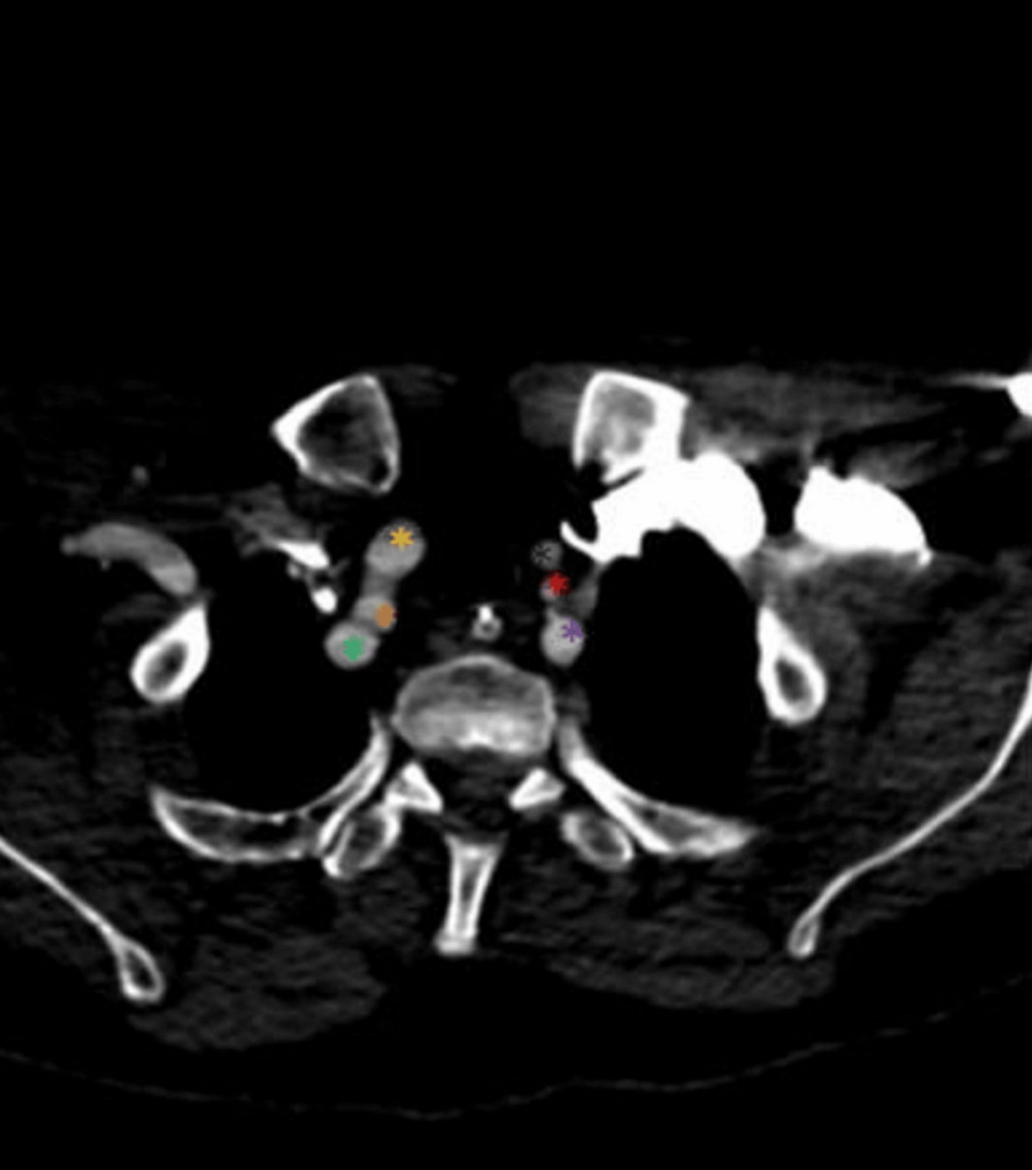
The RVA originates from the BCT and the LVA originates from the AA (axial view) The yellow asterisk marks the RCCA, the orange asterisk marks the RVA, the green asterisk marks the RSA, the black asterisk marks the LCCA, the red asterisk marks the LVA, and the purple asterisk marks the LSA. RVA: Right vertebral artery, LVA: Left vertebral artery, AA: Aortic arch, BCT: Brachiocephalic trunk, RCCA: Right common carotid artery, RSA: Right subclavian artery, LCCA: Left common carotid artery, LSA: Left subclavian artery

**Figure 4:**
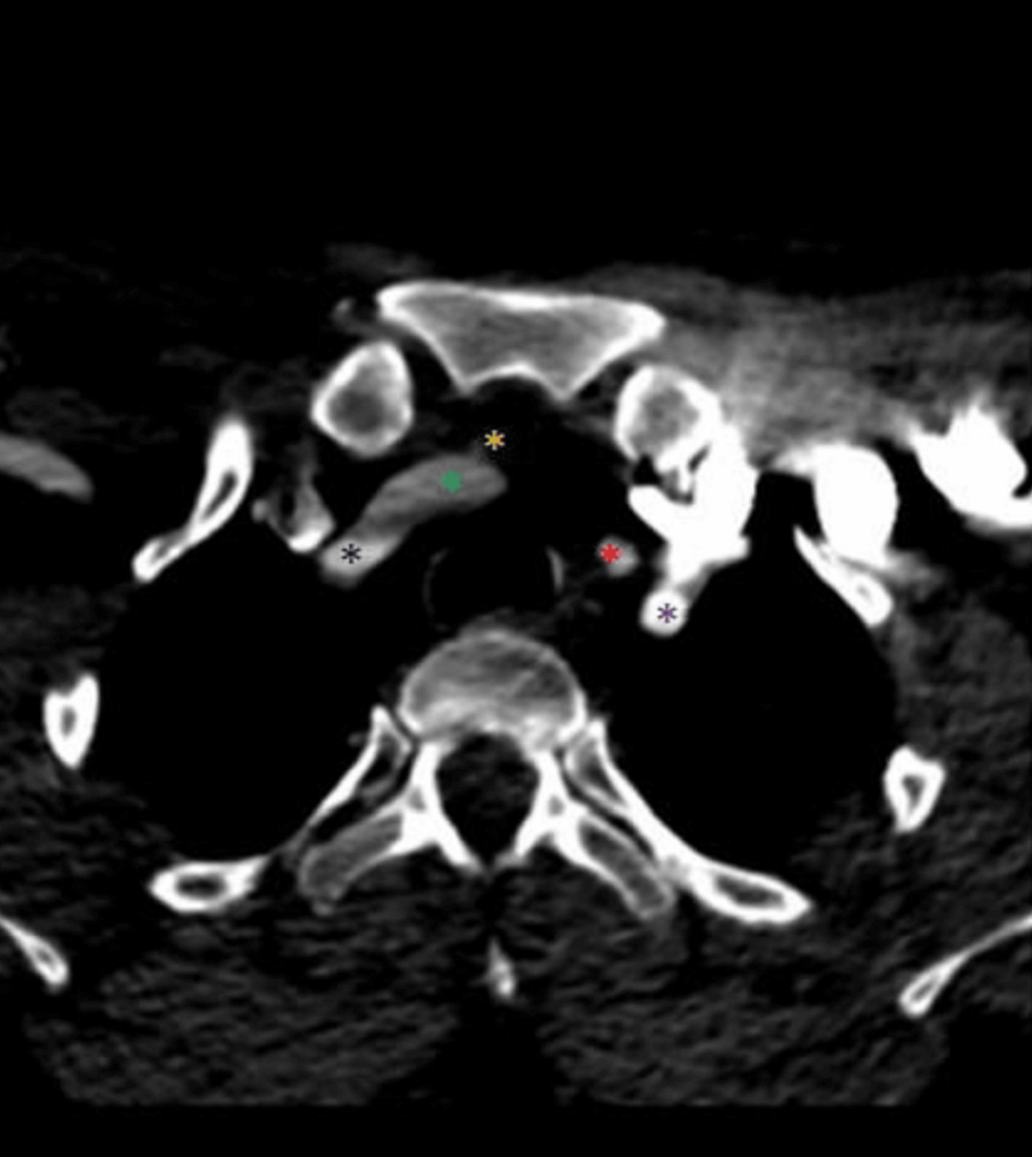
The TIA originates from the BCT (axial view) The yellow asterisk marks the TIA, the green asterisk marks the RCCA, the black asterisk marks the RSA, the red asterisk marks the LCCA, and the purple asterisk marks the LSA. TIA: Thyroid ima artery, BCT: Brachiocephalic trunk, RCCA: Right common carotid artery, RSA: Right subclavian artery, LCCA: Left common carotid artery, LSA: Left subclavian artery

**Figure 5:**
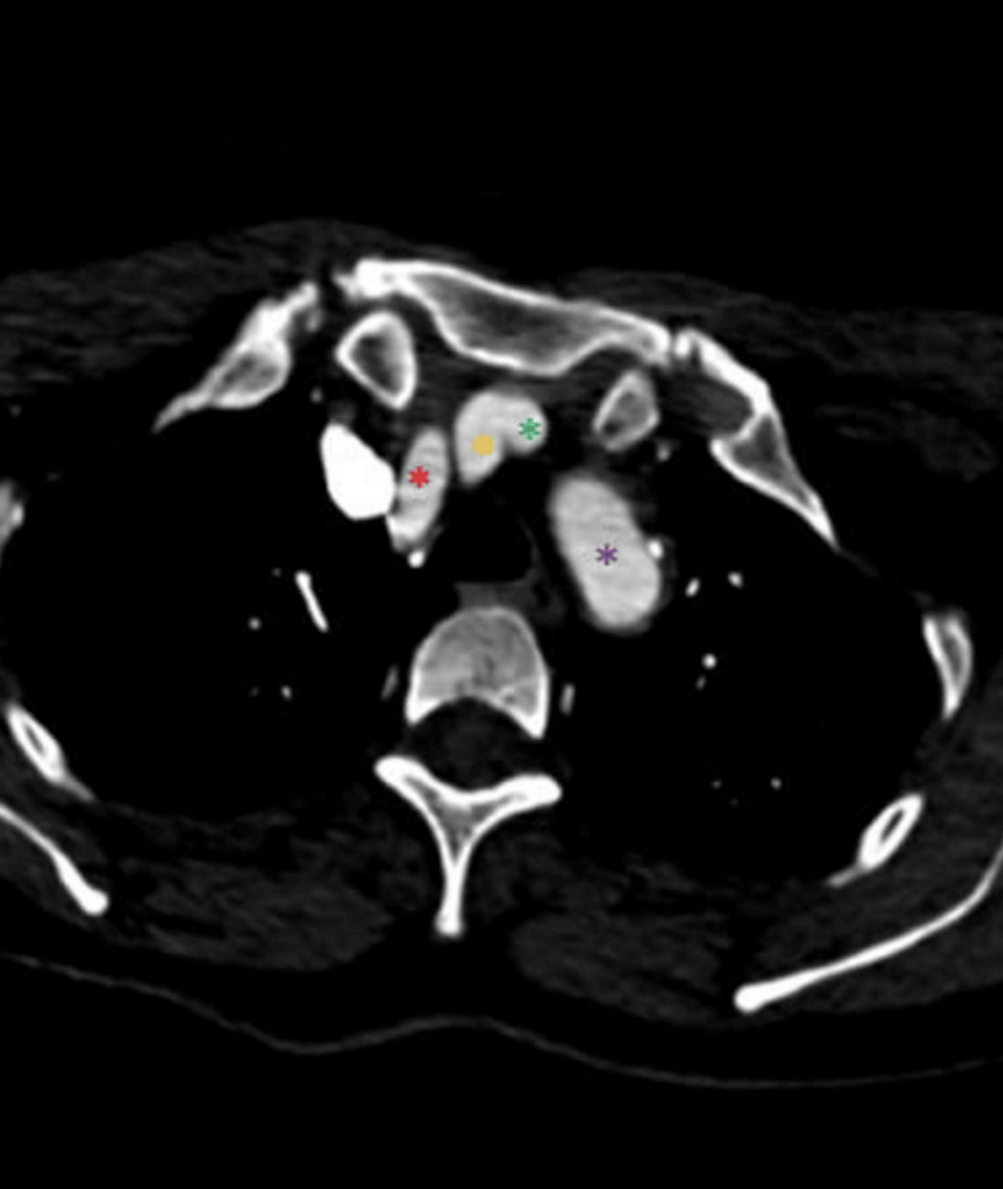
The left and right CCAs originate from the AA as a single trunk (axial view) The yellow asterisk marks the RCCA, the red asterisk marks the RSA, the green asterisk marks the LCCA, and the purple asterisk marks the LSA. CCAs: Common carotid arteries, AA: Aortic arch, RCCA: Right common carotid artery, RSA: Right subclavian artery, LCCA: Left common carotid artery, LSA: Left subclavian artery

**Figure 6:**
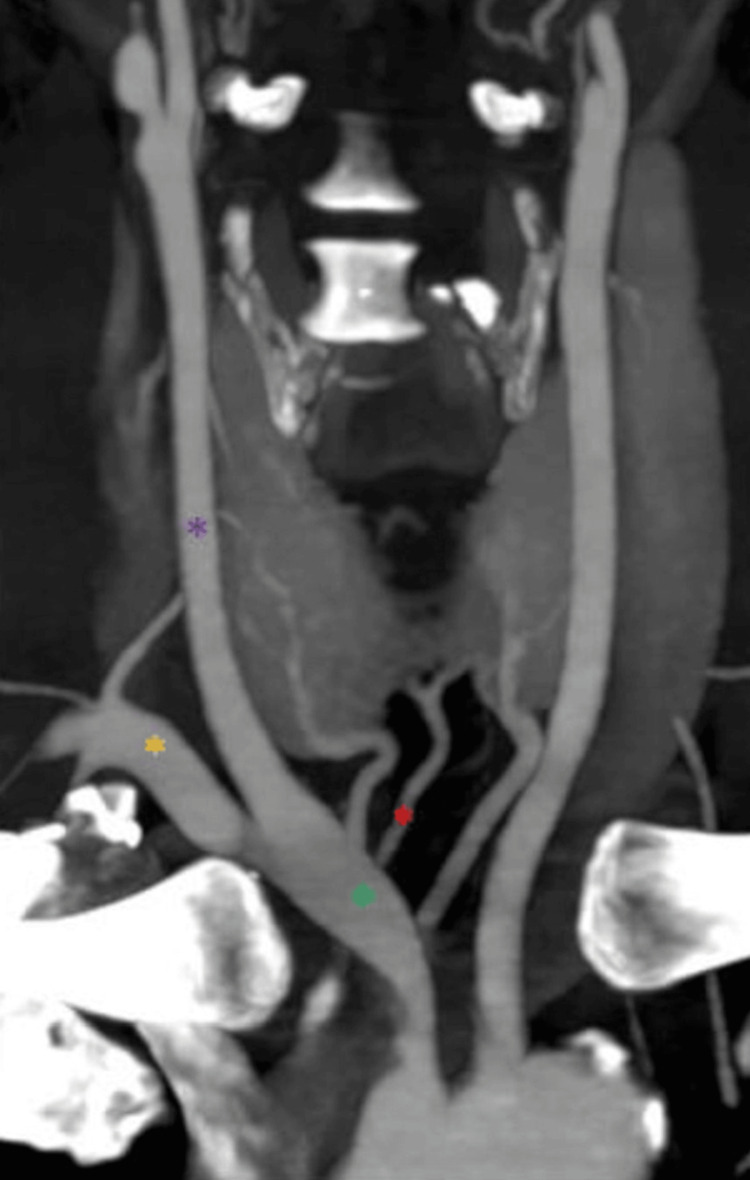
The TIA originates from the BCT (coronal view) The green asterisk symbol marks the BCT, the purple asterisk marks the LCCA, the yellow asterisk marks the LSA, and the red asterisk marks the TIA. TIA: Thyroid ima artery, BCT: Brachiocephalic trunk, LCCA: Left common carotid artery, LSA: Left subclavian artery

According to a systematic review and meta-analysis by Popieluszko et al. [3], seven types of variations are found in the AA (Table 1). Among the 105 participants in the present study, we found that 85 scans (80.95%) were type 1 (normal), while we identified variations in the AA in 20 patients’ CT scans (Figures 1-6). Among these patients, eight had type 2 (bovine arch; 7.61%), five had type 3 (left vertebral; 4.76%), one had type 5 (common carotid; 0.95%), and one had type 7 (right arch AA; 0.95%). 

**Table 1:** Aortic arch variants Variations in the AA as described by Popieluszko et al. [3]. AA: Aortic arch, BCT: Brachiocephalic trunk, LCCA: Left common carotid artery, LSA: Left subclavian artery, LVA: Left vertebral artery, RSA: Right subclavian artery, RCCA: Right common carotid artery

Types	Name	Variation
Type 1	Normal	Right to left - BCT, LCCA, and LSA
Type 2	Bovine arch	Right to left - BCT and LCCA arise as a common stem, LSA
Type 3	Left vertebral	Right to left - BCT, LCCA, LVA, and LSA
Type 4	Bovine and LVA	Right to left - BCT and LCCA arise as a common trunk, followed by LVA and LSA
Type 5	Common carotid	Right to left - RSA, common origin of RCCA, LCC, and LSA
Type 6	Aberrant RSA	Right to left - RCCA, LCCA, LSA, and an aberrant RSA
Type 7	Right arch	Left to right - Releasing a “mirrored” pattern or aberrant LSA

We identified variations (Table 2) in addition to those described by Popieluszko et al. [3]. Thus, two patients had an LCCA originating as a common trunk with the BCT (1.90%), two had a thyroid ima artery (TIA) arising from the BCT (1.90%), and one had a right vertebral artery (RVA) branching from the BCT and an LVA branching directly from the AA (0.95%).

**Table 2:** Distribution of participants in terms of variation in branching pattern (n = 20) LCCA: Left common carotid artery, BCT: Brachiocephalic trunk, LVA: Left vertebral artery, AA: Aortic arch, TIA: Thyroid ima artery, RVA: Right vertebral artery, CCAs: Common carotid arteries

Variation in branching pattern	Frequency	Percentage	95% CI
LCCA originated from the BCT	8	40%	25.2% - 70.5%
LVA originated from the AA	5	25%	10.1% - 51.4%
LCCA and BCT originated from the AA as a single trunk	2	10%	0.3% - 28.1%
TIA originated from the BCT	2	10%	0.3% - 28.1%
LVA originated from the AA; RVA originated from the BCT	1	5%	0.3% - 28.1%
BCT originated as the last left branch from the AA (right arch)	1	5%	0.3% - 28.1%
Right and left CCAs originated as a single trunk from the AA	1	5%	0.3% - 28.1%

The LCCA originated from the BCT in eight patients (40%). The LVA originated from the AA in five patients (25%). The TIA originated from the BCT in 2 patients (10%). The LCCA and BCT originated from the AA as a single trunk in 2 patients (10%). The LVA originated from the AA, and the RVA originated from the BCT in 1 patient (5%). The BCT originated as the last left branch from the AA in one patient (5%). The RCAA and LCCA originated as a single trunk from the AA in one patient (5%).

We used the chi-square test to explore the association between gender and variation in the branching pattern (Table 3). More than 20% of the total number of cells had an expected count of less than five. We observed no significant differences among the various groups in terms of the distribution of variation in the branching pattern (χ2 = 7.769, p = 0.188). The strength of the association between the two variables (Cramer’s V) was 0.64 (strong association). The strength of the association between the two variables (bias-corrected Cramer’s V) was 0.28 (weak association).

**Table 3:** Association between gender (%) and variations in branching pattern of the AA The chi-square test was used to analyse the p-value. LCCA: Left common carotid artery, BCT: Brachiocephalic trunk, LVA: Left vertebral artery, AA: Aortic arch, TIA: Thyroid ima artery, RVA: Right vertebral artery, CCAs: Common carotid arteries

Variation in branching pattern	Gender (n) %			Chi-square Test	
	Male	Female	Total	χ2	p-value
LCCA originated from the BCT	6 (54.6%)	2 (22.2%)	8 (40%)	7.769	0.188
LVA originated from the AA	4 (36.4%)	1 (11.1%)	5 (25%)		
TIA originated from the BCT	1(10%)	1(11.1%)	2 (10%)		
LCCA and BCT originated from the AA as a single trunk	0 (0.0%)	1 (11.1%)	1 (5%)		
LVA originated from the AA; RVA originated from the BCT	0 (0.0%)	1 (11.1%)	1 (5%)		
BCT originated along with the left CCA as a single trunk from the AA	0 (0.0%)	1 (11.1%)	1 (5%)		
BCT originated as the last left branch from the AA	0 (0.0%)	1 (11.1%)	1 (5%)		
Right and left CCAs originated as a single trunk from the AA	0 (0.0%)	1 (11.1%)	1 (5%)		
Total	11 (100.0%)	9 (100.0%)	20 (100.0%)		

We used the chi-square test to analyze the p-value. In the male patients, the LCCA originated from the BCT in 54.6%, the LVA originated from the AA in 36.4%, and the TIA originated from the BCT in 10%. In none of the male patients (0.0%) was the BCT the last left branch from the AA, and in none of the male patients (0.0%) did the LCCA and BCT originate from the AA as a single trunk, the LVA originate from the AA, the RVA originate from the BCT, or the BCT originate along with the LCCA as a single trunk from the AA (Table 3). In the female patients, the LCCA originated from the BCT in 22.2%, the LVA originated from the AA in 11.1%, the BCT was the last left branch from the AA in 11.1%, the LCCA and BCT originated from the AA as a single trunk in 11.1%, the LVA originated from the AA and the RVA originated from the BCT in 11.1%, the BCT originated along with the LCCA as a single trunk from the AA in 11.1%, the TIA originated from the BCT in 11.1%, and the RCCA and LCCA originated as a single trunk from the AA in 11.1%.

Of note, during the follow-up after two months of scans with patients who had variations in the branching pattern of the AA, we found that 15% complained of dyspnea, which can be caused by esophageal or tracheal compression. However, further investigation revealed no other probable cause for the dyspnea.

## Discussion

Kodikara et al. studied the branching pattern of the AA in the Sri Lankan population using CT and identified four forms: type 1 (n = 197, 90%), type 2 (n = 10, 4.6%), type 3 (n = 8, 3.7%), and type 6 (n = 4; 1.8%), with only 10% of the patients showing AA variations [2]. In both genders, type 1 AA was the most common pattern (91% in females, 88.9% in males). For the female patients, type 2 (n = 6; 5.4%) was the most common AA variation, while type 3 (n = 5; 4.6%) was the most common AA variation for the male patients. However, there was no evidence that gender was a significant influence on the AA branching pattern (odds: 0.792; 95% CI: 0.327-1.917; p=0.605). The negative effects of vascular examinations and therapies could be reduced if the frequency of surgically and angiographically significant arch alterations in a given community were known. In the present study, we found five types of branching patterns of the AA, among which 85 patients' (80.95%) scans were type 1 (normal), while there were variations in the AA in 20 patients' CT scans. Among these patients, eight had type 2 (bovine arch; 7.61%), five had type 3 (left vertebral; 4.76%), one had type 5 (common carotid; 0.95%), and one had type 7 (right arch AA; 0.95%). 

Patil et al. studied 75 cadavers in the Nagpur belt of India. They observed the usual three-branched AA in 58 cadavers (77.3%); two branches from the AA, one being the common trunk for the origin of the BCT and the LSA and the second being the origin of the LSA in 11 cadavers (14.66%); and the LVA originating directly from the AA, with the AA branches being (from left to right) the BCT, LCCA, LVA, and LSA in six cadavers (8%) [4]. Understanding the various AA patterns is essential when inserting equipment into this fragile structure and its branches.

Pandalai et al. conducted a study of 4,000 participants from the South Indian population in 2021 to assess anatomical variation in the AA using CT scans (with contrast) [5]. They observed 27 variations of the AA in these patients: seven had an aberrant RSA, one had a bovine arch, one had a bovine origin of the LVA from the AA, one had an anomalous bronchial artery from the AA, one had a double AA, and 16 had a right-sided AA. The prevalence of the variations in the AA observed in their study indicates that imaging should be done before any procedure involving vascular access in order to avoid problems associated with these variations. Further research is necessary to determine the burden of AA variation in the population, particularly with regard to individuals in the Indian subcontinent. These researchers also found that the variations in the AA included two patients in which the LCCA originated as a common trunk with the BCT, two patients in which the TIA originated from the BCT, and one patient in which the RVA branched from the BCT and the LVA branched directly from the AA. In the present study, 85 patients had normal CTAs, while we observed variations in the AA in 20 patients. Of the patients showing variations, eight had a bovine arch, five had the LVA originating from the AA, one had the RCCA and LCCA originating as a single trunk from the AA, and one had a right AA. Additionally, two patients had LCCAs originating as a common trunk with the BCT, two had a TIA originating from the BCT, and one had an RVA branching from the BCT and an LVA branching directly from the AA.

Aboulhoda et al. used CTA to study variations in the AA in the Egyptian population in 2019 and found that the bovine arch was the most common abnormal branching pattern, occurring in 24% of the patients [6]. Of these patients with bovine arch, 6% had the common ostium variant, and 5% had an aberrant LVA emerging directly from the AA. These researchers argued that, for patients undergoing aortic endovascular intervention, the anatomical variations and morphometric data are crucial information for selecting the size, shape, and type of angiographic catheters and devices to be supplied.

Mustafa et al. conducted a CTA study on the branching patterns of the AA in the Jordanian population in 2017 and found six artery arrangements based on the origin of arteries from the AA [7]. The typical classical pattern was the most prevalent, being observed in 61.2% of the patients. The six AA variations were seen in the remaining patients (38.8%). No significant relationship was found between gender and the prevalence of AA variations. These researchers provided fresh data regarding the frequency of variations in AA branching among Jordanians, including the numerous structural differences in the branching patterns. These findings should, therefore, be taken into account when angiography, aortic instrumentation, or supra-aortic thoracic, head, and neck surgery are performed.

In a cadaveric study of the variations in the branching patterns of the AA in the North Maharashtra region, Shinde et al. found that 59 cadavers (89.39%) had three-branched AAs; five (7.57%) had only two branches, one of which was a common trunk that included the LCCA, BCT, and LSA; and the LVA originated directly from the AA in two cadavers (3.33%) [8].

Karacan et al. conducted a CTA study of the AA branching patterns of 1,000 Turkish patients in 2014 [9]. They found that 79.2% of the patients had a normal branching pattern (type 1) and 20.8% had variations in the AA. Specifically, 14.1% had the type 2 branching pattern (the BCT and LCCA originating as a common trunk from the AA); 4.1% had the type 3 pattern (the LVA originating from the AA); 1.2% had the type 4 branching pattern (the coexistence of type 2 and type 3); 0.6% had the type 5 pattern (aberrant RSA); 0.7% had the type 6 branching pattern (the coexistence of an anomalous RSA and a bicarotid trunk); and 0.1% had the type 7 branching pattern (the TIA originating from the AA). The incidence of AA branching variation was similar in men and women (20.0% and 22.1%, respectively). It is critical to recognize abnormalities in the branching of the AA because they may result in symptoms resulting from compression of the tracheoesophageal tube, complications from surgery, or endovascular interventions involving the aorta and its branches. Interruption of the embryological right fourth AA between the carotid and subclavian arteries causes this subclavian artery abnormality.

Goldsher et al. conducted a retrospective study to determine the prevalence of bovine AAs in 417 human fetuses between the 15th and 40th weeks of gestation in 2019 [10]. Throughout the study, 417 fetuses were analyzed, and an AA was detected in 413 cases (99%, trial group). In the remaining four cases, poor resolution and an unfavorable fetal position made it impossible to identify the AA. With a range of 15 to 40 weeks, the mean gestational age was 26 weeks. Twenty of the 413 individuals in whom an AA was detected had the bovine arch variation (4.8%, 95% CI 3.1-7.3%). There was no significant difference between the median gestational age of diagnosis of bovine arch (28 + 3; IQR 23 + 4-31 + 5) and controls (26 + 0; IQR 22 + 3-19 + 6; p =.098). In 103 individuals, fetal abnormalities were found, including 77 mild alterations and 26 significant malformations. Goldsher et al. found that 20 of 413 fetuses (4.8%, 95% CI, 3.1-7.3%) had a bovine arch, 14 of 310 (4.5%) had no fetal abnormalities, and six of 77 (7.8%) showed slight alterations (p = 0.241). None of the 26 fetuses with significant abnormalities had the bovine arch.

Ramasamy and Ramasamy conducted a study of 50 adult human cadavers (42 male and eight female) over five years (2013 to 2018). They found that all of the cadavers had a left-sided AA and observed normal branching in 41 [11]. Among the variations in the other nine cadavers, five had a common trunk for the brachiocephalic and LCCAs, two had four branches, including an extra origin of the LVA, one had three branches, specifically, a common trunk (brachiocephalic + LCCA) and the LVA and LSA, and one had four branches, including an additional RSA.

Berko et al. conducted a study of anomalies in the thoracic vasculature and found that the most prevalent AA abnormality was an atypical RSA. The frequency of this anomaly in their study was 1.2%, while the frequency in two other investigations (DeGaris and Klinkhamer) was 0.4% [12] and 2% [13]. In the study by Berko et al., the abnormal RSA typically ran retroesophageally. Although an abnormal RSA was once believed to cause the clinical condition known as dysphagia lusoria, this abnormality is typically asymptomatic and an accidental discovery. Early detection of an abnormal RSA is necessary when preparing for esophageal and vascular surgical and angiographic operations [14]. In the present study, too, patients complained about dysphagia.

Since our study involved a single center, it suffers from the limitations that only 105 participants could be recruited and that they were recruited from a narrow geographical region, specifically the Garhwal region of the sub-Himalayan belt. The images were taken using 65 or 128-slice images, which may have lower spatial resolution. Fewer slices may miss fine anatomical details, leading to less accurate diagnosis and increased risk of artifacts, and thicker slices can cause partial volume effects, blurring small lesions or structures. To overcome these limitations and expand the implications for clinical practice, a multicenter study is needed involving participants from a broad geographical region.

## Conclusions

Variations in the branching pattern of the AA are quite common and have widespread clinical implications, from planning endovascular interventions to executing cardiothoracic surgeries. We observed variations in the branching pattern of the AA in 19.5% of the participants in our study. Such variations are a major concern for surgeons, for failure to notice them may result in serious complications. To improve the accuracy of surgical and interventional radiological head and neck treatments and reduce the associated morbidity, healthcare workers (surgeons and radiologists) must be aware of the variations in the branching pattern of the AA. Currently, a comprehensive preoperative CTA examination of the neck and thorax is essential for precise surgical planning as well as averting potential complications. The best patient management practice during neck surgery requires an understanding of the intricate anatomy of AA branching. Prior to surgery, most patients are assessed using cross-sectional imaging modalities, among which CTA may be especially useful in this situation owing to its noninvasive nature.
